# 

**Published:** 1888-08

**Authors:** 


					﻿NOTICE.
All articles for publication, books for review, business communications, etc.,
must be sent to Editors, 1123 Spruce Street, Philadelphia.
Subscribers will please notice that this Journal will be sent until arrears
are paid and it is ordered discontinued.
Am I my brother’s keeper ? ” You are to the extent of
being negligent of his best interests if you let him, be igno-
rant of the great work THE HOJMLCEOPATHICPHYSI-
CIAN is doing. See that your friends know of this work.
				

